# Donor Hematopoietic Stem Cells Confer Long-Term Marrow Reconstitution by Self-Renewal Divisions Exceeding to That of Host Cells

**DOI:** 10.1371/journal.pone.0050693

**Published:** 2012-12-05

**Authors:** Sushmita Roy, Saleem Javed, Swatantra K. Jain, Subeer S. Majumdar, Asok Mukhopadhyay

**Affiliations:** 1 Stem Cell Biology, National Institute of Immunology, New Delhi, India; 2 Department of Biochemistry, Jamia Hamdard, New Delhi, India; 3 Department of Biotechnology, Jamia Hamdard, New Delhi, India; 4 Cellular Endocrinology, National Institute of Immunology, New Delhi, India; Emory University, United States of America

## Abstract

Dormant hematopoietic stem cells (HSCs) are activated by microenvironmental cues of the niche in response to the injury of bone marrow (BM). It is not clearly understood how engrafted cells respond to these cues and are involved in marrow regeneration. The purpose of this study was to decipher this cellular response in competitive environment. BM cells of CD45.2 mice were transplanted in sub-lethally irradiated CD45.1 mice. The status of the donor and recipient stem cells (LSK: Lin^−^Sca-1^+^c-Kit^+^) were determined by flowcytometry using CD45 alleles specific antibodies. The presence of long-term engraftable stem cells was confirmed by marrow repopulation assay in secondary hosts, and cell cycle status was determined by staining with Ho33342 and pyronin Y, and BrdU retention assay. The expressions of different hematopoietic growth factor genes in stromal compartment (CD45^−^ cells) were assessed by real-time reverse transcriptase- polymerase chain reaction (RT-PCR). The presence of donor cells initially stimulated the proliferation of host LSK cells compared with control mice without transplantation. This was expected due to pro-mitotic and anti-apoptotic factors secreted by the donor hematopoietic cells. Upon transplantation, a majority of the donor LSK cells entered into cell cycle, and later they maintained cell cycle status similar to that in the normal mouse. Donor-derived LSK cells showed 1000-fold expansion within 15 days of transplantation. Donor-derived cells not only regenerated BM in the primary irradiated host for long-term, they were also found to be significantly involved in marrow regeneration after the second cycle of irradiation. The proliferation of LSK cells was associated with the onset of colossal expression of different hematopoietic growth factor genes in non-hematopoietic cellular compartment. Activation of donor LSK cells was found to be dynamically controlled by BM cellularity. Long-term study showed that a high level of hematopoietic reconstitution could be possible by donor cells in a sub-lethally irradiated host.

## Introduction

Osteoblastic niche maintains long-term hematopoietic stem cells (LT-HSCs) in quiescent (G_0_) state [1]–[5]. Stem cells remain attached to the niche cells through many cell surface molecules [6], [7]. Niche protects HSCs from myelo-suppressive stresses. Dormant or quiescent HSCs are activated and undergo self-renewal, following asymmetric division of cells, in response to any stress or stimulation with granulocyte colony-stimulating factor (G-CSF) [8]. Self-renewal division of HSCs generates a large number of transiently amplified progenitors and matured cells for replenishment of the loss of bone marrow (BM) cellularity. Once marrow regeneration is completed, activated HSCs return back to dormancy [8].

It has been reported that in response to the combination treatment of cyclophosphamide (CY) and G-CSF, the endogenous HSCs proliferate about 10-fold prior to the mobilization in the peripheral blood [9], [10]. In another study, the repopulation ability of HSCs was shown to be significantly improved by treatment of mice with G-CSF and stem cell factor (SCF) [11]. The rapid expansion of HSCs following above treatments suggested that most of these cells had entered in the cell cycle [9]. In management of hematological malignancy, often combination of radiation and chemotherapy is given to the patients, which may severely affect the hematopoietic system. This may affects other vital organs also. The hematopoietic system is reconstituted by transplanting BM cells, especially HSCs. How these donor HSCs respond to the ablated BM environment is not clearly understood. Earlier studies showed that in humans as well as in mouse, bone marrow chimera eventually fails in the long run. This could happen due to either or combination of (a) rapidly dividing donor HSCs become defective in engraftment on osteoblastic niche, (b) osteoblastic niche looses control over donor-HSCs due to competition with endogenous cells for the space, thus these cells are engrafted in the vascular niche and slowly egress from the marrow environment after differentiation, and (c) asymmetric self-renewal property of donor HSCs is lost. The divisions of LT-HSCs and short term (ST)-HSCs are considered to be related with the cell cycle status, capable of long-term and short-term engraftment potential, respectively. The quiescent LT-HSCs are responsible for long-term engraftment; whereas cells exit from the G_0_ phase (ST-HSCs/multipotent progenitors) are engrafted for the short-term [12]–[14]. The difference between these cells was linked with marrow homing capacity. However, a recent study showed that ST-HSCs are also capable for long-term multilineage engraftment in an irradiated host [15].

In this study, we showed that there is reversibility between dormancy and proliferation of donor HSCs during marrow regeneration. In the competitive environment, host cells initially proliferated; later were found to be compromised in the presence of highly proliferating donor cells. We also report that mouse BM stromal cells transiently expressed thousands to million folds of hematopoietic growth factor genes compared to normal mouse stromal cells. This induction of growth factor genes was commenced with the proliferation of HSCs.

## Materials and Methods

### Animals

Eight- to ten-week-old C57BL/6J [*Ptprc^b^* (Ly5.2)] and BL6.SJL [*Ptprc^c^* (Ly5.1)] mice were used in this study. Mice were kept in an isolator, and fed with autoclaved acidified water and irradiated food *ad libitum*. All procedures were approved by the Animal Ethics Committee (No. 112/07), National Institute of Immunology, New Delhi, India.

### Hematopoietic Reconstitution

BM cells were isolated in two stage process from 6- to 8-week-old CD45.2 mice. In the first step, cells were removed from tibia and femurs by flushing with medium. The firmly adhered cells of the endosteal region were recovered in the second stage by digesting the crushed bone with collagenase type IV (0.03%) and dispase enzyme (2 U/ml). BM cells were pooled in a tube and erythrocytes were lysed by treatment with Gey’s solution [16]. Three different doses (2.5, 5, and 10×10^6^ per mice) of cells were transplanted in sub-lethally (700 cGy) irradiated CD45.1 mice through lateral tail vein injection. Further, in another experiment 3×10^4^ sorted CD45.2LSK cells were transplanted in each mouse, as above. Sorting of cells was done with a customized FACSAriaIII (BD Biosciences) using 70-µm nozzle. Mice were sacrificed at different time intervals for analysis.

### Competitive Marrow Repopulation Assay

After 10 and 15 days of transplantation, primary (1°) recipients were sacrificed and BM cells were isolated as described above. Donor cells (CD45.2) from 1° mice were purified by a one-step magnetic activated cell sorting (MACS) technique. In brief, BM cells were incubated with biotinylated CD45.2 antibody (eBiosciences, San Diego, CA) for 15 min at 8°C. Excess antibody was washed out, resuspended cells in MACS buffer were further incubated with streptavidin microbeads (Miltenyl Biotec, Bergisch, Gladbach, Germany) for 15 min at 8°C. Finally, washed magnetically labelled cells were positively sorted in a LS column. The purity of the cells was determined by negative staining with anti-CD45.1/PECy5. Four doses (10, 30, 100, 300×10^3^) of sorted cells were transplanted in the secondary (2°) host (CD45.1). After 30 days of transplantation donor-derived LSK cells were analyzed.

### Cell-cell Interaction Study

This study was conducted by both *in vitro* and *in vivo* experiments. *In vitro* experiments were conducted in two ways: (i) recipient and donor cells were co-cultured (contact) in 6-well plates, and (ii) cells were cultured by separating them (non-contact) using 3-µm cell culture insert (Millicell, Millipore Corporation, Billerica, MA) in 24-well plates. In co-culture experiments, 2×10^6^ irradiated (700 cGy) BM cells of CD45.1 mouse were cultured with same number of unirradiated CD45.2^+^ cells in 2 ml of medium (IMDM supplemented with 5% ES-certified FCS). In control experiments, CD45.2^+^ cells were not used. In non-contact experiments, 0.5×10^6^/300 µl irradiated CD45.1-mouse BM cells were taken in the lower chamber, whereas 0.2×10^6^/300 µl of unirradiated CD45.2^+^ cells were taken on the insert and cultured. The control experiments were devoid of CD45.2^+^ cells. After day 1 and 2, cells in the lower chamber were analyzed for apoptosis by staining with CD45.1 and Annexin V (Apoptosis Detection Kit, BD Pharmingen) antibodies. Another fraction of cells were fixed with 4% paraformaldehyde for 30 min, and then permeabilized using 0.1% saponin. The washed cells were stained with CD45.1 and Ki67 specific antibodies (Santa Cruz Biotechnology, Santa Cruz, CA) to determine the proliferation of host cells. Finally, the labelled cells were analyzed with a customized FACSAriaIII (BD Biosciences) using specific band-pass filters.

*In vivo* studies were conducted by transplanting 3×10^4^ sorted CD45.2LSK cells in each mouse. Mice without and with transplantation were sacrificed on day 3, 5 and 10 for analysis of necrotic (PI staining) and apoptotic (Annexin V staining) cells of CD45.1LSK compartment. Furthermore, these cells were stained with anti-cyclin A antibody (Santa Cruz) for immunocytochemical analysis. Cells were also subjected to real-time RT-PCR analysis for *p21* expression.

### Cell Cycle Analysis

Recipient- and Donor-derived LSK cells were subjected to cell cycle analysis by staining with Hoechst 33342 (Ho) and Pyronin Y (PY) (Sigma-Aldrich, St Louis) dye [17]. Staining of cells was done by incubating 1×10^6^ cells/500 µl of 1% FCS containing IMDM with 5 µl Ho dye (1 mg/ml) at 37°C for 1 h. Cells were washed, resuspended in the same medium and further stained with PY (1.6 µg/ml) by incubating at 37°C for 1 h. Ho-PY stained cells were washed twice and labelled for LSK prior to analysis.

### BrdU Pulse-chase

Post-transplanted mice were given BrdU pulse for 10 days. Initially, one injection (100 mg/Kg body weight) was given through intra-peritoneal route, which was followed by feeding (1 mg/ml) through drinking water [18]. The pulse was chased for 20 days by feeding normal drinking water to the mice. Mice were sacrificed at 10 and 30 days of transplantation, BM cells were harvested and BrdU incorporations in donor and recipient LSK cells were determined by flow-cytometric analysis. Prior to the staining, LSK cells were fixed in 4% paraformaldehyde for 30 min, followed by denaturation with 2 N HCl containing 0.5% Triton X-100 for 15–20 min. Washed cells were stained with anti-BrdU IgG/FITC antibody (BD Biosciences, San Jose, CA).

### Immuno Histochemistry (IHC)

Engraftment of donor stem cells on the endosteal niche was identified by immunohistochemical analysis. GFP-expressing crude BM cells were transplanted to 700 cGy irradiated C57BL6/J mice. After 30 days of transplantation, mice were sacrificed and tibia and femurs were isolated. Bones were fixed in 4% PFA and decalcified in 5% formic acid. Five micron bone tissue cryosections were stained with mouse anti-GFP (Clonetech, CA, Mountain View), mouse osteopontin (Santa Cruz), and anti-Sca-1 (eBioscience) antibodies. Alexa Fluor 488/594/555 secondary antibodies (Molecular Probes, Inc., Eugene, OR) were used to identify the specific proteins. The nuclei were stained with 4′,6-diamidino-2-phenylindole (DAPI). Sections were imaged with a Zeiss LSM 510 META confocal laser-scanning microscope using a Plan-Apochromat 63×/1.4 oil objective. LSM 510 software was used for acquisition of images. The images were processed by Zeiss LSM Image browser, version 4.2.0.121.

### Flowcytometry

Cells were stained with antibodies Sca-1/FITC, CD45.2/APC, CD45.1/PECy5, c-Kit/APC (eBisciences), biotinylated lineage antibody cocktail (Miltenyl), c-Kit/PECy5, Flk2/APC (BD Pharmingen) for 30 min on ice. Cells were washed three times with phosphate buffered saline (PBS) containing 0.5% bovine serum albumin (BSA). The lineage cocktail antibody-stained cells were further incubated with streptavidin-APCCy7 (BD Pharmingen) for an additional period of 30 min. Washed cells were analyzed with a customized FACSAriaIII (BD Biosciences) using specific band-pass filters.

### Real-time RT-PCR

The methods for the extraction of total RNA and synthesis of cDNA are provided in Method S1. Primers for real-time RT-PCR were designed using Primer Express software version 2.0 (Applied Biosystems, Foster City, CA). The sequences of the primers and the conditions of the reactions are given in Table S1.

### Statistical Analysis

Results of multiple experiments are reported as the mean ± standard error of the mean (SEM). One-way ANOVA was used to calculate the significance between the two experimental groups.

## Results

### Donor Derived LSK Cells are Predominantly Amplified during Marrow Regeneration

**Figure 1:**
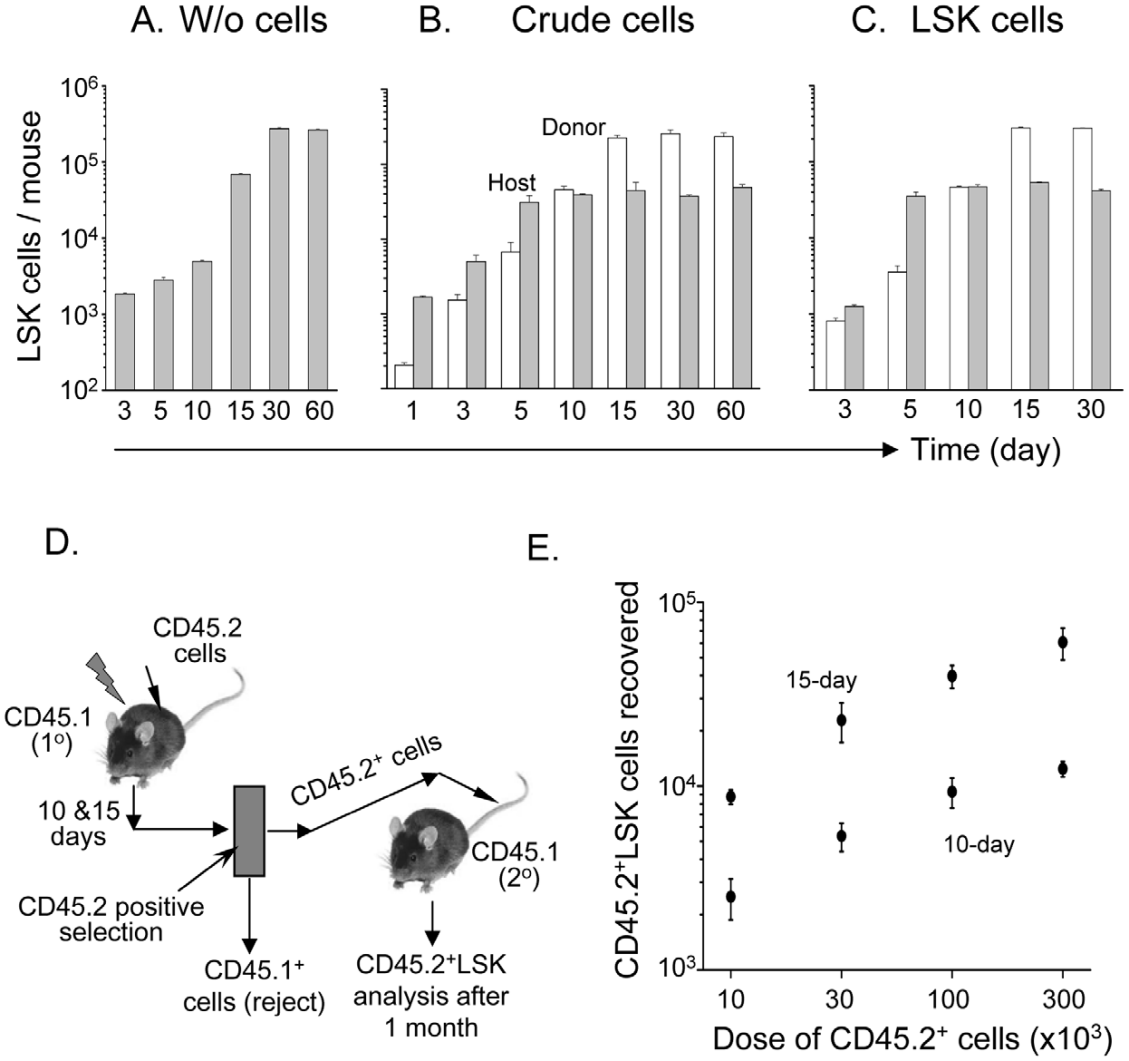
Proliferation of LSK cells during marrow regeneration. (A) Sub-lethally irradiated mice were maintained (without transplantation) for different time points. Mice were sacrificed and LSK cells were determined by flowcytometry. Bar diagram shows proliferation kinetics of host BM-LSK cells (n = 6, each time point); (B) Each sub-lethally irradiated mouse received 10×10^6^ crude BM cells of CD45.2 mouse. Mice were sacrificed and LSK cells were determined by flowcytometry. Bar diagram shows proliferation kinetics of host and donor LSK cells (n = 6, each time point); (C) Each sub-lethally irradiated mouse received 3×10^4^ CD45.2LSK cells. Mice were sacrificed and LSK cells were determined by flowcytometry. Bar diagram shows proliferation kinetics of host and donor LSK cells (n = 3, each time point); (D, E) Competitive marrow repopulation assay. Donor hematopoietic (CD45.2^+^) cells of 10 and 15 days of transplantation were isolated from primary recipient. Four different doses of above sorted cells (10×10^3^, 30×10^3^, 100×10^3^ and 300×10^3^) were transplanted in 4 groups of mice, each consisting of 4. After 1 month of transplantation, BM cells were isolated from secondary recipient and analyzed for donor-derived LSK cells. Proportional increase in the recovery of donor LSK with transplantation dose are shown in E.

In order to examine the proliferation kinetics of donor-derived LSK cells and possible competition with that of host, crude BM cells (CD45.2) were transplanted in irradiated hosts (CD45.1). We observed increase of LSK cells in BM with the increase of cell dose (Figure S1). However, early detection of donor LSK cells (within 24 h of transplantation) was made possible at a dose of 10×10^6^ cells. Henceforth, all experiments using crude cells were conducted using the same dose. To study of host cells-derived hematopoiesis in non-competitive environment (without transplantation), a group of CD45.1 mice were irradiated and maintained for 2 months. Mice were sacrificed at different time intervals, and BM cellularity and total LSK cells were determined. About 120-fold increase of LSK cells between 3^rd^ and 30^th^ day of irradiation was observed (Fig. 1A). In contrast, no expansion of host LSK cells was noticed (with transplantation) after 5^th^ day of transplantation. The expansion of these cells was prominent only between 1^st^ and 5^th^ day of transplantation, with 15-fold increase in number (Fig. 1B). In contrast, the donor-derived LSK cells were steadily increased up to 15^th^ day of transplantation, about 1000-fold amplification was observed (Fig. 1B). These results suggest that donor cells, at the beginning of transplantation induced proliferation of host LSK cells, which later entered into quiescent stage. To know whether donor LSK cells serve the same functions as seen in case of crude cells, we transplanted equivalent number of stem cells (see materials and methods) and mice were sacrificed at different time intervals. Analyses of total recovered host and donor LSK cells showed that the results were comparable with crude donor cells (Fig. 1B and 1C). The host LSK cells initially showed increase in number, whereas later 5^th^ day onwards no proliferation was observed. Interestingly, in this case also donor LSK cells proliferated till 15^th^ day of transplantation (Fig. 1C).

To ensure that LSK cells are nothing but engraftable HSCs, a competitive marrow repopulation assay (MRA) was conducted for CD45.2^+^ cells, isolated from 1° recipient, in 2° recipients. Initially, CD45.2^+^ cells were harvested on 10^th^ and 15^th^ day post-transplantation from 1° recipients by positive selection method (Fig. 1D). The sorted cells were transplanted in 2° hosts at four different doses (10,000 to 300,000 cells per mouse), and CD45.2LSK cells were analyzed after a month of transplantation. The purpose of using different doses of cells was to show that with increase of cell number, HSCs (LSK) populations were proportionately increased and hence the engraftment. Higher the number of HSCs in 1° hosts greater will be the engraftment and LSK cells recovery from 2° hosts. It was found that marrow repopulation, in terms of donor LSK cells, was increased with cell dose (Fig. 1E). Further, similar trend of results was observed in samples of both 10^th^ and 15^th^ day, and LSK cells were found to be several folds higher in the later time point in all 4 doses of cells. Combining these results, it may be concluded that donor-derived HSCs were more in number in 15^th^ day than 10^th^ day of engraftment.

### Cell Cycle Activation of Donor LSK Cells is Dynamically Controlled

**Figure 2:**
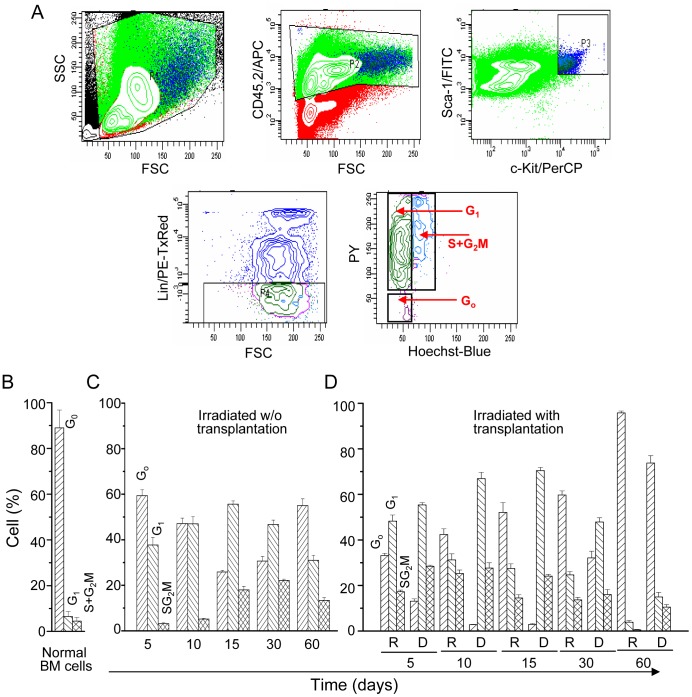
Cell cycle status of LSK cells during marrow regeneration. Cells were stained for LSK, followed by Ho+PY. (A) A representative dot-plot analysis of the donor cells. Donor-derived LSK cells were selected as follows: donor cells (CD45.2^+^) were first selected, followed by gating for Sca-1^+^c-Kit^+^ and Lin^−^ Sca-1^+^c-Kit^+^ (LSK) cells. Finally, CD45.2^+^LSK cells were analyzed for Ho and PY. For recipient cells, instead of CD45.2, cells were stained with CD45.1 antibody. (B) Bar diagram shows cell cycle status of LSK cells in healthy mice (n = 4); (C) Bar diagram shows cell cycle status of host LSK cells after irradiation (n = 6, each time point); (D) Bar diagram shows cell cycle status of recipient and donor LSK cells during marrow regeneration (n = 6, each time point).

In the osteoblastic niche, HSCs are maintained in quiescent (G_0_) state. Soon after irradiation, marrow HSCs enter into cell cycle to reconstitute bone marrow. To determine the kinetics of cell cycle activation of recipient and donor HSCs and competition between these cells, if any, respective LSK cells were stained with Ho and PY. Ho is a DNA inter-chelating dye commonly used for cell cycle analysis, whereas PY binds with both RNA and DNA. In the presence of Ho, PY binds specifically with RNA [17]. To know cell cycle status, crude BM cells were gated for donor- derived LSK cells (Fig. 2A). Similar gating was also applied for host LSK cells (not shown). In the G_0_ state the cellular activity is minimum, which is increased in the metabolically active (G_1_) state of the cells. Thus, Ho_lo_-stained diploid LSK cells were divided into two parts, one that stained low or no with PY (truly representing G_0_ cells) and the other that stained high PY (representing G_1_ cells), as shown in the contour plot (Fig. 2A, lower right). In irradiated mice (without transplantation), G_0_ cells were significantly (*p*<0.05) dropped to 25.85±2.68% (n = 6) within 15 days, before it tend to regain the normal value (Fig. 2B and 2C). These results confirmed the entry of host LSK cells into the cycle for proliferation and reconstitution of hematopoietic system.

**Figure 3:**
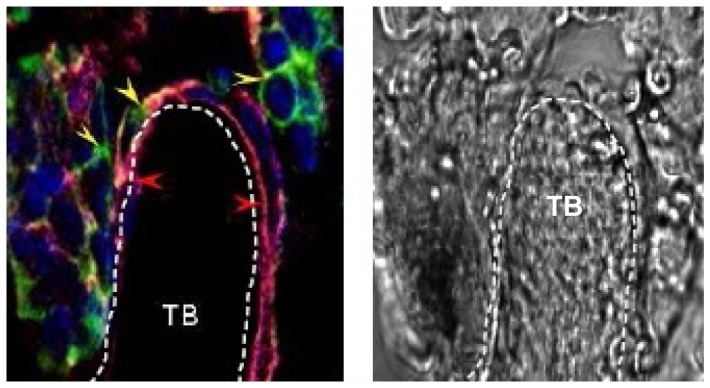
Donor-derived hematopoietic progenitor cells are localized in the trabicular bone. Sub-lethally irradiated mice were transplanted with GFP-expressing crude BM cells. After a month, mice were sacrificed; longitudinal trabicular bone cryosections (5 µm) were obtained. Sections were stained for osteopontin (AF594, red), GFP (AF488, green), Sca-1 (AF555, pink) and nuclei (DAPI, blue). Both immunofluorescence (composite) and bright filed confocal images are shown. Arrows indicating donor derived progenitor cells.

In irradiated mice (with transplantation), the situation was different. The G_0_ fraction of donor LSK cells was declined and remained low up to 15^th^ day, which later steadily increased till the end of the study (Fig. 2D). This was due to competition between the donor and the host cells in which host cells were compromised as a result of radiation and/or donor cells naturally activated as they were removed from their niche. This was concluded from the cell cycle status of the donor-LSK cells, in which G_0_ cells were declined from 87.8±8.5% (normal BM) to the lowest values of 2.5±0.45% (n = 6) in day 10 (Fig. 2D). The activation of donor-HSCs was accompanied by rapid proliferation of the cells as shown in Figure S2A and S2B **.** One most interesting observation made from this study was that like host, the activity of donor LSK cells might have controlled by the stem cell niche. Soon after the partial recovery of BM, donor LSK cells gradually entered into quiescent state, perhaps by lodging on the osteoblastic niche. Immuno-histochemical analysis of the trabicular bone confirmed that a good number of donor-derived stem cells were present near osteoblastic niche area after 30 days of transplantation (Fig. 3). Overall, it was surmised that highly proliferating donor LSK cells become dormant by targeting to the osteoblastic niche.

### Transplantation Rescues Host Cells from Apoptosis and Induces their Proliferation

**Figure 4:**
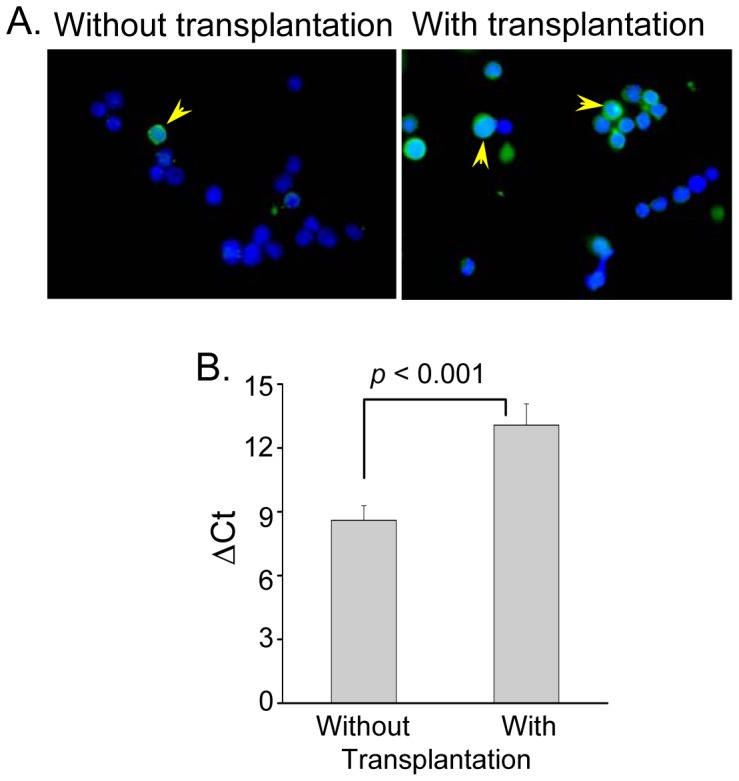
Expression of cyclin A and *p21* ^cip1/waf1^ in CD45.1LSK cells. (A) Sub-lethally irradiated CD45.1 mice were transplanted with or without CD45.2LSK Cells. Mice were sacrificed after 3 days of transplantation and host LSK cells were sorted and immuno- stained for cyclin A protein. Representative images show that a few cells expressed cyclin A (arrow) in untransplanted mouse than transplanted (magnification 60×); (B) Host LSK cells of above two groups were compared for the expression of *p21* gene by real time RT-PCR. Bar diagram shows relative ΔCt values in two different conditions (n = 3).

In the previous experiments, recovery of the host LSK cells in the initial few days of transplantation was significantly high as compared to mouse that does not received donor cells. We hypothesized that donor cells might have some role in this process. To decipher that, mice with and without transplantation were sacrificed and host cells (CD45.1LSK) were analyzed to know relative apoptotic and necrotic cells. Representative dot-plots of flowcytometry analyses are shown in Figures S3 and S4, and the compiled data are presented in Table 1. In each of the three times, percentage apoptotic and necrotic cells was significantly (*p*<0.001) declined in case of mice received transplantation. These observations suggest that donor cells contributed some humeral effect to host LSK cells for salvaging some of them from undergoing apoptosis. Further, to confirm relative cell cycle activation in these cells we analyzed cyclin A and G_1_/S-check point regulation cyclin-dependent kinase inhibitor *p21* ^cip1/waf1^ expressions. The cyclin A protein was found to be expressed in larger fraction of cells in mice received transplantation than without the transplantation (Fig. 4A). This result has been supplemented with *p21* mRNA expression, which declined significantly in case of transplantation (Fig. 4B). Above studies envisage two specific roles of donor cells: (a) recovery of host LSK from undergoing apoptosis, and (b) activation of their proliferation.

**Table 1:** Anti-apoptotic effect of normal donor cells on irradiated host cells.

	Apoptotic CD45.1LSK cells (Annexin V^+^ in %), m ± sem		Necrotic CD45.1LSK cells(PI^+^ in %), m ± sem	
Time (days)	Without transplantation	With transplantation	Without transplantation	With transplantation
3	26.60±2.40^*^	8.20±0.36^*^	45.60±1.70^*^	28.20±1.50^*^
5	18.06±1.30	3.40±0.37	46.30±1.70	8.70±0.60
10	10.90±0.58	4.10±0.75	8.50±0.70	1.20±0.14
Significance		^*^ *p*<0.001		^*^ *p*<0.001

Number of mice in each time point was 3 to 4.

*In vitro* studies were conducted to know whether cell-cell direct interaction is necessary to confer above protective and proliferative effects. Irradiated host cells were cultured with direct and indirect contact of CD45^+^ donor cells. It may be seen that the proliferation of host cells was significantly (*p*<0.001) increased on day 2 of culture (Table 2) in the presence of donor cells, as more cells stained for nuclear protein (Ki67). Similarly, apoptotic cells in the host compartment was significantly (*p*<0.05) declined on day 1 of culture in the presence of donor cells, as fewer cells stained for Annexin V. Further, analysis of the results revealed that direct cellular contact may not be required for these early beneficial effects on irradiated host cells as non-contact experiments performed equally well (Table 2). Overall, results of the above experiments implicated some soluble factors, secreted by donor hematopoietic cells, responsible for increase in proliferation of CD45.1LSK cells in the initial few days of transplantation.

**Table 2:** Effect of normal hematopoietic cells on proliferation and anti-apoptotic effects in irradiated bone marrow cells in culture (direct or indirect contact).

	Proliferation (Ki67^+^ cells %), m ± sem				Apoptosis (Annexin V^+^ cells %), m ± sem			
	Direct contact		Indirect contact		Direct contact		Indirect contact	
Time (days)	W/o donor cells	With donor cells	W/o donor cells	With donor cells	W/o donor cells	With donor cells	W/o donor cells	With donor cells
0	2.6±0.10	2.6±0.10	2.6±0.10	2.6±0.10	5.4±0.25	5.4±0.25	5.4±0.25	5.4±0.25
1	2.4±0.05	9.1±0.06	1.6±0.10	7.5±0.05	24.5±4.50^*^	7.3±1.45^*^	19.7±2.15	8.3±0.30
2	10.2±0.15^*^	20.2±0.90^*^	10.5±0.10	32.0±0.50	14.3±1.75	7.5±1.35	13.0±0.30	7.8±1.20
Significance		^*^ *p*<0.001		*p*<0.001		^*^ *p*<0.05		*p*<0.03

Above values were calculated from flowcytometric analyses of respective samples (n = 2 to 3). Representative dot-plots of flowcytometric analyses are shown in Figures S5 and S6.

### Donor LSK Cells Retain High Proportion of BrdU at 30 Days of Pulse-chase

**Figure 5:**
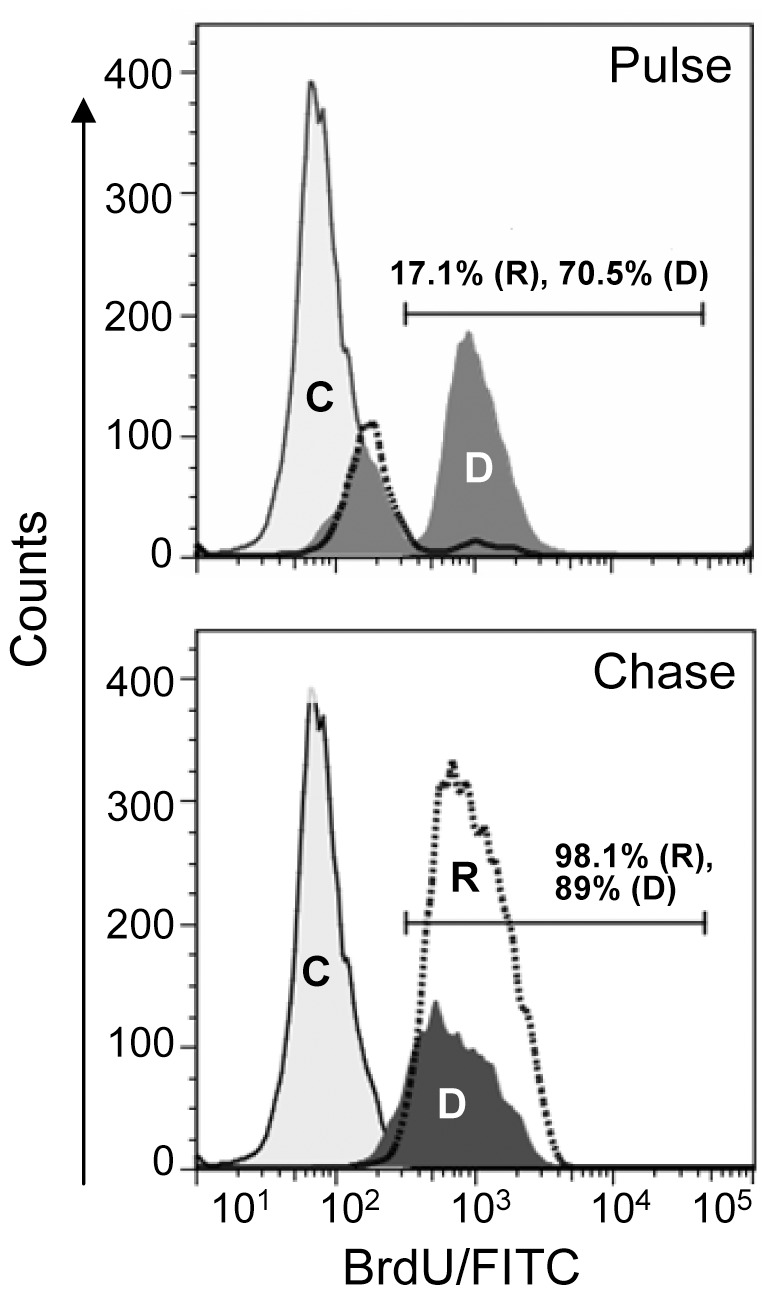
Pulse-chase experiment for nuclear incorporation of BrdU. After transplantation as above, a group of mice were given BrdU pulse for 10 days (see Materials and Methods), which was followed by chase for 20 days. First LSK cells were sorted and then stained for BrdU. The representative histogram (top) show 70.5% of donor (D) and 17.1% of recipient (R) LSK cells were labelled with BrdU. The representative histogram (bottom) shows 98% of recipient and 89% of donor cells retained BrdU. C: Control LSK cells (n = 3).

To confirm that donor stem cells entered much faster in cell cycle than host, we performed BrdU pulse-chase experiments. Mice were given long pulse of BrdU for 10 days followed by 20 days of chase. The duration of pulse was maintained long as LT-HSCs are known to be slowly entered into cell cycle [19]. Results during pulse showed 68±9% donor LSK cells incorporated BrdU, whereas in the same time only 20±4.5% host LSK cells incorporated the same analogue (Fig. 5, top). These results suggest that relatively a small fraction of host LSK cells were in the cell cycle during the period of pulse. This could be due to two possible reasons: (1) a large fraction of host LSK cells remain in quiescent state as they adhered on the marrow niche, and/or (2) they received radiation injury, which prevented entry of cells in to the S-phase. To know the proportion of BrdU retaining cells in both the compartments, a 20 days pulse-chase was conducted. Interestingly, all host and majority of the donor-derived LSK cells retained BrdU, though a modest dilution effect was noticed in case of donor LSK cells (Fig. 5, bottom). Furthermore, almost complete exhaustion of BrdU^-^LSK cells were observed in both host and donor compartments during the chase period.

### Donor LSK Cells Maintain High Marrow Turnover Efficiency

**Figure 6:**
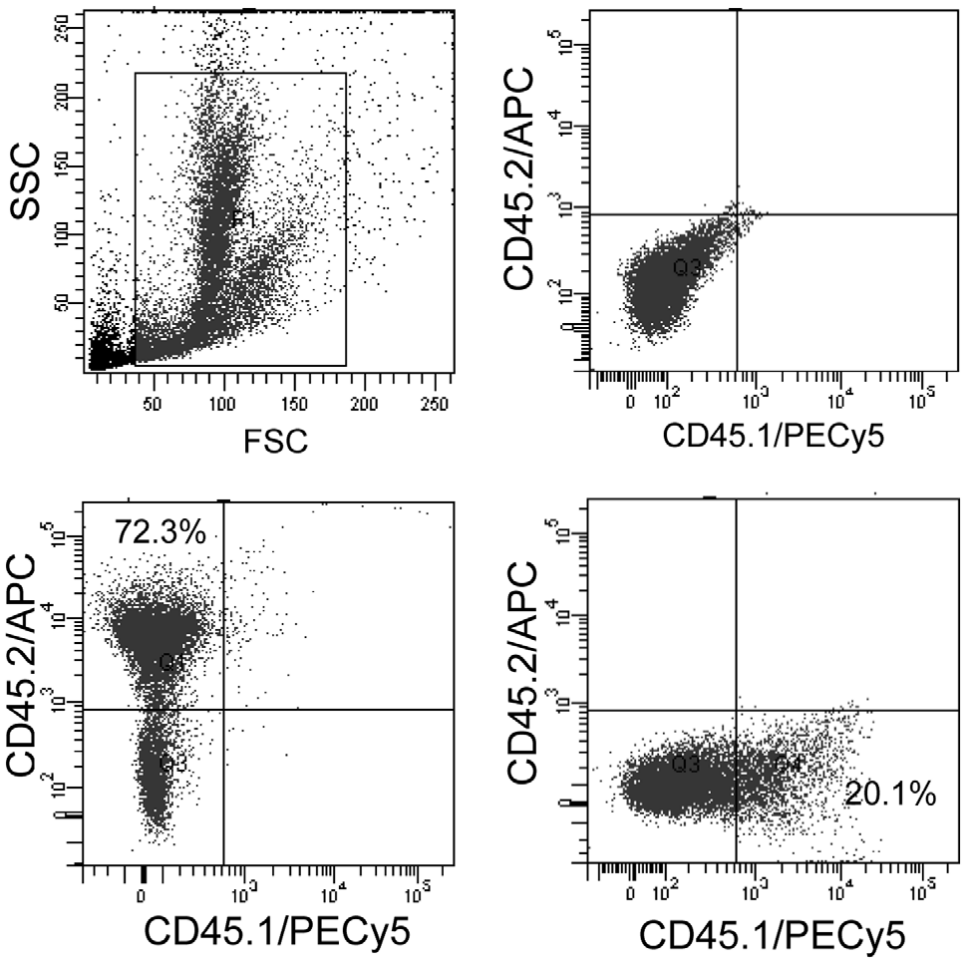
BM donor cells chimerism in irradiated mice. Experiment was initiated as before, after 7 months of transplantation mice were subjected to second cycle of irradiation and maintained for 1 month. Mice were sacrificed and BM cells were analyzed for chimerism. Representative dot-plot show 78% donor (CD45.2) cells chimerism (n = 3).

In order to show that donor-derived HSCs can maintain marrow regeneration capacity even after irradiation, we transplanted donor cells into sub-lethally irradiated mouse. Donor cells chimerism was routinely analysed till 7 months of transplantation, it was 76.4±6.1% (Figure S7). Further, to confirm the capability of the donor cells for marrow regeneration, these mice were exposed to radiation for the second time. After a month, each mouse was examined for BM chimerism with respect to donor (CD45.2) cells. All four mice showed high degree (76.8±7.4%) of donor cells chimerism (Fig. 6). BM cells were further analyzed for LT-HSCs and ST-HSCs, results suggested no significant difference (percent) between donor and host in these two classes of stem cells of (Table 3). However, significantly (*p*<0.05) higher number (absolute) of donor-derived stem cells was recovered compared to host (Table 3).

**Table 3:** HSC population in bone marrow after second cycle of irradiation.

HSCs	Host LSKFlk2^+^ cells	Host LSKFlk2^−^ cells	Donor LSKFlk2^+^ cells	Donor LSKFlk2^−^ cells
%	0.033±0.006	0.008±0.003	0.165±0.042	0.043±0.014
Absolute number	1464±309*	331±125	24360±5446* (*p*<0.05)	6853±2278 (*p*<0.05)

The absolute numbers of HSCs were calculated by multiplying % stem cells with total cells recoverfed.

* ^,¶^Significantly higher as compared to the same phenotype of the host cells.

### Hematopoietic Growth Factor Genes are Highly Expressed in Early Stromal Compartment

**Figure 7:**
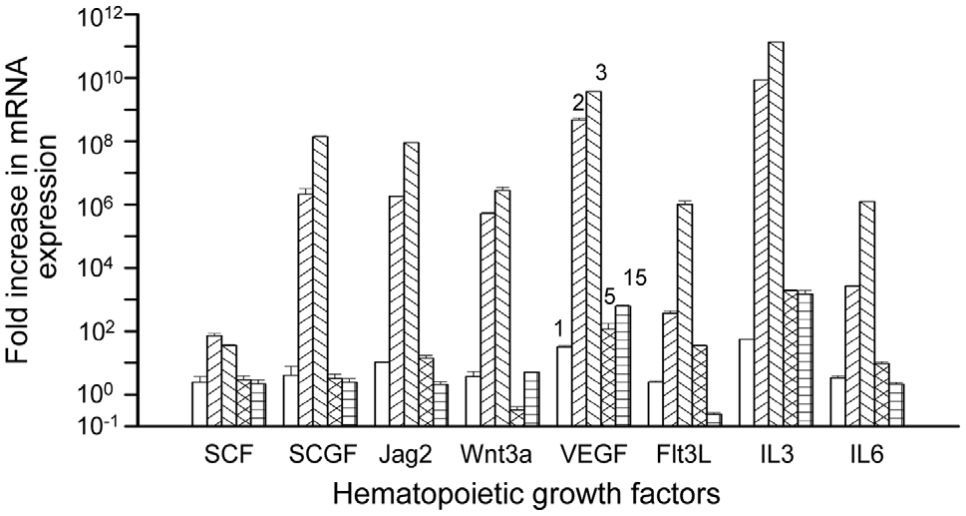
Kinetics of gene expression by real time RT-PCR. BM cells were harvested at different time points following irradiation and transplantation; stromal cells (CD45^−^) were sorted and analyzed for relative (fold) expression of the hematopoietic growth factor genes compared to that in unirradiated and untransplanted mice. Bar diagrams show fold expressions of mRNAs for different growth factors at days 1, 2, 3, 5 and 15 of transplantation (n = 3).

The above results prompted us to examine gene expressions of different hematopoietic growth factors/cytokines that support self-renewal of HSCs. Since stromal cells express these factors, CD45^−^ cells were sorted from mice at different days of transplantation for real-time RT-PCR analyses of stem cell factor (SCF), fetal liver tyrosine kinase ligand (Flt3L), stem cell growth factor (SCGF), Jagged 2 (Jag2), wingles-type MMTV integration site family members 3A (Wnt3a) ligand, vascular endothelial growth factor (VEGF), interleukin-3/6 (IL-3/6) genes. The results showed similar pattern of expression for above genes, having peak levels at day 2 and 3 (Fig. 7). After that the expressions of genes were significantly (*p*<0.05) reduced from day 5 onwards. These results probably indicated that high expressions of these growth factors may not be required after 5 to 10 days of regeneration. Further, it was revealed that despite stiff decrease of gene expression from day 3, transient amplification of LSK cells continued till day15 of transplantation (Fig. 1B and Fig. 7).

## Discussion

Bone marrow transplantation (BMT) is generally practiced in cases of non-malignant and malignant hematological disorders [20]. The purpose of transplantation is to reconstitute/rejuvenate hematopoietic system for continuous supply of blood lineages and to induce graft versus leukemic (GVL) reaction (in case of leukaemia). Though HSC is the major player to induce physiological changes in the recipients, the cellular environment is found to play a critical role for engraftment, proliferation and differentiation of the cells. In the present study we showed that, under a competitive environment, donor LSK cells proliferate much rapidly than the host during marrow regeneration. Cell cycle status suggested that there exist a reversible switch in regenerating marrow, which induces quiescent stem cells into actively proliferating state and then bring some of them back into quiescent state.

Most of the clinical transplantation protocols use high-dose total body irradiation (TBI) to prevent rejection of the donor graft [21], to create a condition (by increasing vascular permeability) in which stem cells are able to home to the BM niches [22], [23]. Further, high dose of TBI allows engraftment of cells by creating more space in the bone marrow or minimizing competition between remnant host stem cells and infused donor stem cells [24], [25]. Our earlier study showed that degeneration of stroma can cause poor retention of donor cells in BM of lethally irradiated mice [26]. Therefore, in this study mice were conditioned by sub-lethal dose of irradiation. As observed in this study, transient amplification of host-derived LSK cells within first few days of transplantation was significantly improved to that in control mice received no graft. In contrast, at later time points a suppressive effect was seen in host compartment, probably due to competition between two classes of cells for the niche, one that was exposed to radiation and the other not. Interestingly, we showed that soluble factors, secreted by donor hematopoietic cells, could able to rescue some host cells undergoing apoptosis and also induce their proliferation. As a result of that, the net effect was increase in cell number. This was supported by the expression of two critical cell cycle regulators. Cyclin A accumulates at the G_1_/S check point entry, and associated with cdk2/cdk1 cyclin- dependent kinases [26], [27]. It’s accumulation at the sites of DNA replication suggesting active involvement in DNA synthesis. The expression of cyclin A in the larger population of host LSK cells, recovered from the mouse received transplantation, ensures that donor cells enhanced cell cycle activation. Irradiation, like several therapeutic agents, induces DNA damage resulting growth arrest of the cells. In response to DNA damage, p53 transcriptionally activates the cyclin-dependent kinase inhibitor p21 and in turn inhibits cell proliferation for allowing DNA repair [28]. In HSCs, p21 maintains quiescence; conversely decline/absence of p21 has been marked with the proliferation and increase in absolute number of cells [29]. The stiff decline in *p21* expression suggested that radiation-exposed host LSK cells might have recovered much early from DNA repair path and thus experienced faster proliferation in the presence of donor cells than the absence. We propose that early entry in cell cycle was induced by some soluble factors secreted by donor cells. At present the identity of these soluble factors are not know, we speculation that they are different from the factors secreted by the stromal cells. It is needless to mention that at least in transcription level stromal-derived factors were abundant in host compartment. Earlier, it was shown that human peripheral blood leucocytes conditioned media can provide protection to rat cardiomyocytes in culture against necrosis and apoptosis, and a down-stream regulator of Wnt pathway ‘WISP1’ was one of the factors expressed by the cells [30]. It was discernable from our preliminary experiments that *WISP1* expresses higher amount in unirradiated than irradiated cells (data not shown). In a report, normal hematopoietic cells secreted IL-12 was found to confer cytoprotective effect to HSCs against irradiation [31].

Other interesting features of this study were enormous expansion of donor-derived LSK cells and achieving normal BM cellularity. Previous reports showed that treatment of mice with CY and G-CSF leads to 10-fold proliferation of endogenous HSCs, prior to the mobilization of cells in the peripheral blood [9], [10]. This study did not evidence mobilization of cells in the peripheral blood (data not shown), despite massive expansion of stem and progenitor cells was noticed. It may be remembered that the present study dealt with marrow regeneration by the engrafted cells, whereas above studies [9], [10] were concerned with egression and mobilization of cells in the peripheral blood. A competitive marrow repopulation assay was used for assessment of the functional HSCs in the graft [32]. The results of MRA not only ensured that HSCs amplified in the primary recipient, but also indicated that they were enriched in terms of stemness and/or engraftability. For same dose of transplantation, the recovery of donor’s LSK cells was 3 to 5 folds higher in day 15 than in day 10. This is the first experimental evidence to demonstrate that symmetric self-renewal divisions of some donor HSCs might have occurred to regenerate depleted marrow cells following radiation injury. Symmetric division of stem cells was reported in other tissues. For example, in rodent forebrain, increase in symmetric cell divisions was observed after the stroke [33]. However, it is generally believed that under steady-state conditions (during normal physiological processes), stem cells divide asymmetrically.

Quiescent HSCs are maintained in the osteoblastic niche [1], [2]. In the course of asymmetric division they are believed to come out from the niche, and once the division process is completed the daughter stem cells return to the niche milieu [34]. The stiff drop in quiescent (G_0_) cells with concomitant increase in G_1_ and SG_2_M phase cells suggested that most of the donor LSK cells were actively proliferating in first 15 days of transplantation. Later, with increase in BM cellularity, these cells gradually withdrew from cell cycle and entered in to the quiescent state. The presence of donor-derived stem/progenitor cells in the trabicular bone area corroborated this observation. Earlier it has been shown that host-derived HSCs can enter into active state from dormancy and vice versa, under the influence of hematopoietic stress 35. BrdU is incorporated during DNA replication undividing cells consistently retain BrdU in the nuclei. Since under steady-state conditions stem cells rarely divide, nuclear retention of BrdU confirms quiescence of the cells. The results of pulse-chase experiments supported our earlier notion that majority of the donor-derived LSK cells were in active proliferation state. Further, it was revealed that both in the donor and in the recipient compartment a commendable fraction of LSK cells retained BrdU; these results did not conclude that all labelled cells were in quiescence state. Despite a greater fraction of label retaining LSK cells were present in the host compartment, as compared to that in donor compartment. The results after the second exposure of irradiation confirmed that donor LSK cells can confer high marrow turnover efficiency in this irradiation model.

Marrow regeneration by donor and host stem cells following irradiation not only depends on the number of short-term and long-term HSCs, microenvironmental cues also have a pivotal role [36]. On the basis of microarray results (data not shown), we selected eight hematopoietic growth factors and cytokines for their gene expressions in early stage of marrow regeneration. *In vitro* culture showed that these factors alone or in combination induces self-renewal of mouse and human primitive stem and progenitor cells, and also prevents apoptosis [20], [37]–[40]. Although the expression of gene does not always correlates with the synthesis of corresponding protein, we assumed that like mRNA there may be transient expression of growth factors by the stromal cells. These results perhaps suggest that the presence of these growth factors at higher concentrations is required for activation of stem and progenitor cells. Once cells are activated their fate is decided by the intrinsic factors. One interesting observation was made that stem cell growth factor (SCGF) gene, previously known as Clec11a, highly expressed at the initial phase of marrow regeneration along with other hematopoietic growth factor genes. Earlier studies showed that SCGF gene was highly expressed in human, rat and mouse stromal cells [41]. Whether SCGF can independently act on primitive stem cells is not apparent, but this and previous [41] results indicating that it may have a link with the primitive HSCs. *In vitro* experiments showed that SCGF can potentiate the effect of many cytokines for expansion of human CD34^+^Lin^−^ or CD34^+^CD38^−^ cells [42]. Our study also showed a differential effect of stromal cells-derived cytokines on donor and host LSK cells in terms of cellular proliferation and differentiation, the reason of which is not clearly understood.

BMT is practiced in different routine and in many experimental therapies. The ultimate success of this therapy is depending on the long-term hematopoietic reconstitution and negligible or no GVHD. This study enriches our knowledge on proliferation status of donor HSCs in a competitive environment. Overall, we conclude that combination of sub-lethal dose of irradiation and high dose of donor cells would be beneficial for reconstituting bone marrow with respect to donor cells.

## Supporting Information

Figure S1 **Effect of cell dose on the recovery of LSK cells.** Each group of mice were transplanted with three different doses of cells (2.5, 5.0 to 10×10^6^). Donor- and recipient-derived LSK cells were analyzed after 2 months of transplantation by flowcytometry. Bar diagram represents the results of 5 animals in each group.
(TIF)

Figure S2 **Distribution of donor, recipient and stromal cells.** Sub-lethally irradiated mice were left untransplanted (A) or transplanted (B) with crude BM cells (10×10^6^/mice). A. Bar diagram shows distribution of hematopoietic and stromal compartment of cells. Hematopoietic (CD45.1^+^) cells and stromal (CD45.1^−^) cells were analyzed by flowcytrometry. The mean values were plotted (n = 6); B. Bar diagram shows distribution of hematopoietic and stromal compartment of cells. Hematopoietic (CD45.1^+^, CD45.2^+^) and stromal (CD45.1^−^CD45.2^−^) cells were analyzed by flowcytrometry. The mean values were plotted (n = 6).
(TIF)

Figure S3 ***In vivo*** **cytoprotection of irradiated host cells.** Sub-lethally irradiated mice were left untransplanted (A) or transplanted (B) with 3×10^4^ CD45.2LSK cells. Mice were sacrificed 3, 5 and 10 days of transplantation and host LSK cells were analyzed for apoptotic cells by staining with Annexin V. Representative dot-plots are shown.
(TIF)

Figure S4 ***In vivo*** **cytoprotection of irradiated host cells.** Sub-lethally irradiated mice were left untransplanted (A) or transplanted (B) with 3×10^4^ CD45.2LSK cells. Mice were sacrificed 3, 5 and 10 days of transplantation and host LSK cells were analyzed for necrotic cells by staining with PI. Representative dot-plots are shown.
(TIF)

Figure S5 ***In vitro*** **proliferation of irradiated host cells.** Sub-lethally irradiated host (CD45.1) cells were cultured in the absence (control) or in the presence (test) of unirradiated CD45.2^+^ cells in contact or without contact. The host cells were analyzed for Ki67 staining by flowcytomtery.
(TIF)

Figure S6 ***In vitro*** **cytoprotection of irradiated host cells.** Sub-lethally irradiated host (CD45.1) cells were cultured in the absence (control) or in the presence (test) of unirradiated CD45.2^+^ cells in contact or without contact. The host cells were analyzed for Annexin V staining by flowcytomtery.
(TIF)

Figure S7 **Donor cells chimerism.** Mice were transplanted with 10×10^6^ crude donor (CD45.2) cells. Chimerism was determined seven months of transplantation. Dot-plot analyses show chimerism of donor (CD45.2) and host (CD45.1) cells for three mice (n = 3).
(TIF)

Table S1 **Real-time primers sequences, amplicon sizes, annealing temperatures and cycle number of PCR reactions.**
(DOC)

Method S1 **Real-time RT-PCR.**
(DOCX)
