# Supplementary figures and images for: Donor Hematopoietic Stem Cells Confer Long-Term Marrow Reconstitution by Self-Renewal Divisions Exceeding to That of Host Cells
Source: PLoS One. 2012 Dec 5;7(12):e50693. doi: 10.1371/journal.pone.0050693 (PMC3515605; doi:10.1371/journal.pone.0050693)

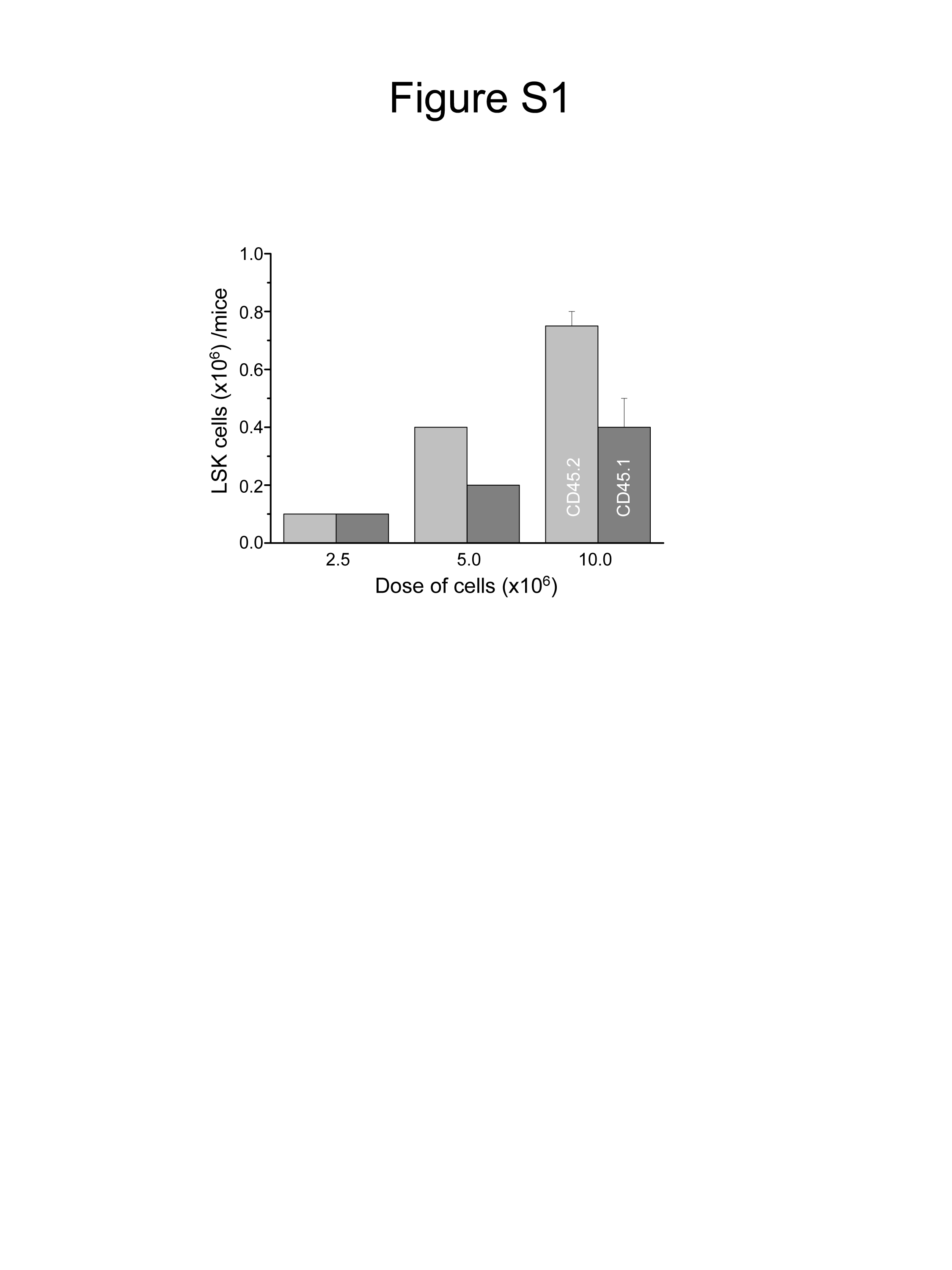

Supplement: Figure S1 — Effect of cell dose on the recovery of LSK cells. Each group of mice were transplanted with three different doses of cells (2.5, 5.0 to 10×106). Donor- and recipient-derived LSK cells were analyzed after 2 months of transplantation by flowcytometry. Bar diagram represents the results of 5 animals in each group. (TIF) [file pone.0050693.s001.tif]

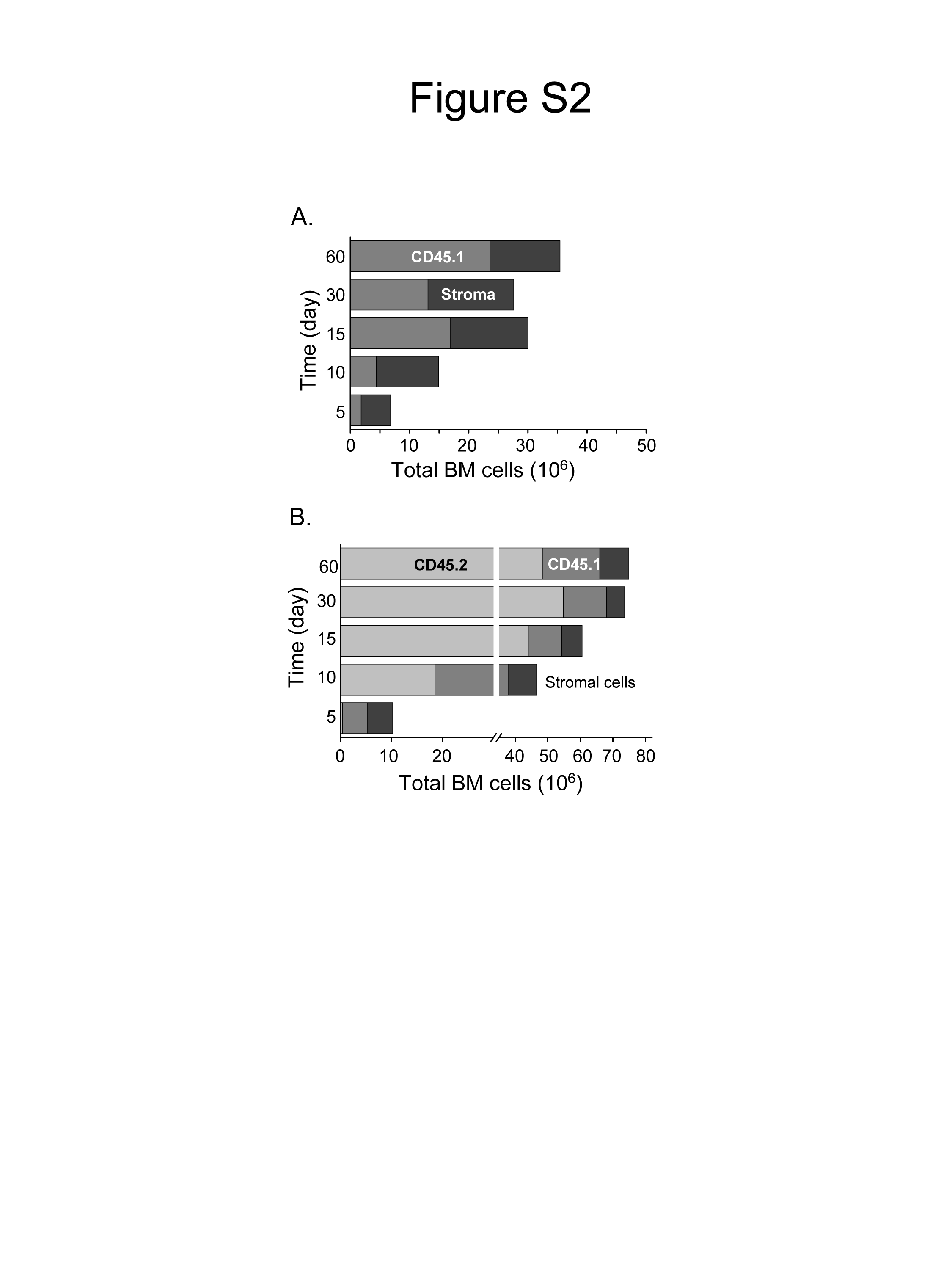

Supplement: Figure S2 — Distribution of donor, recipient and stromal cells. Sub-lethally irradiated mice were left untransplanted (A) or transplanted (B) with crude BM cells (10×106/mice). A. Bar diagram shows distribution of hematopoietic and stromal compartment of cells. Hematopoietic (CD45.1+) cells and stromal (CD45.1−) cells were analyzed by flowcytrometry. The mean values were plotted (n = 6); B. Bar diagram shows distribution of hematopoietic and stromal compartment of cells. Hematopoietic (CD45.1+, CD45.2+) and stromal (CD45.1−CD45.2−) cells were analyzed by flowcytrometry. The mean values were plotted (n = 6). (TIF) [file pone.0050693.s002.tif]

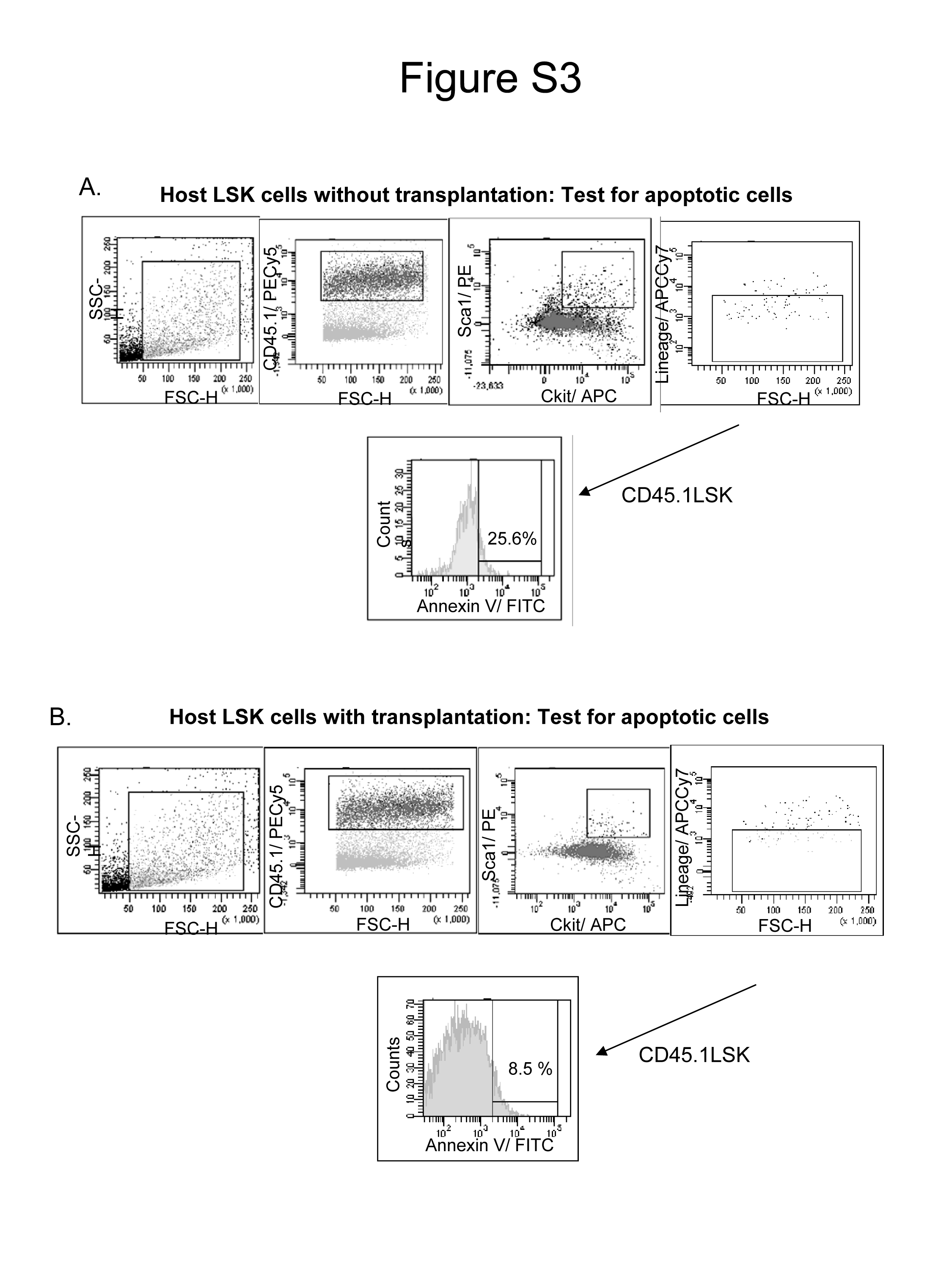

Supplement: Figure S3 — In vivo cytoprotection of irradiated host cells. Sub-lethally irradiated mice were left untransplanted (A) or transplanted (B) with 3×10^4^ CD45.2LSK cells. Mice were sacrificed 3, 5 and 10 days of transplantation and host LSK cells were analyzed for apoptotic cells by staining with Annexin V. Representative dot-plots are shown. (TIF) [file pone.0050693.s003.tif]

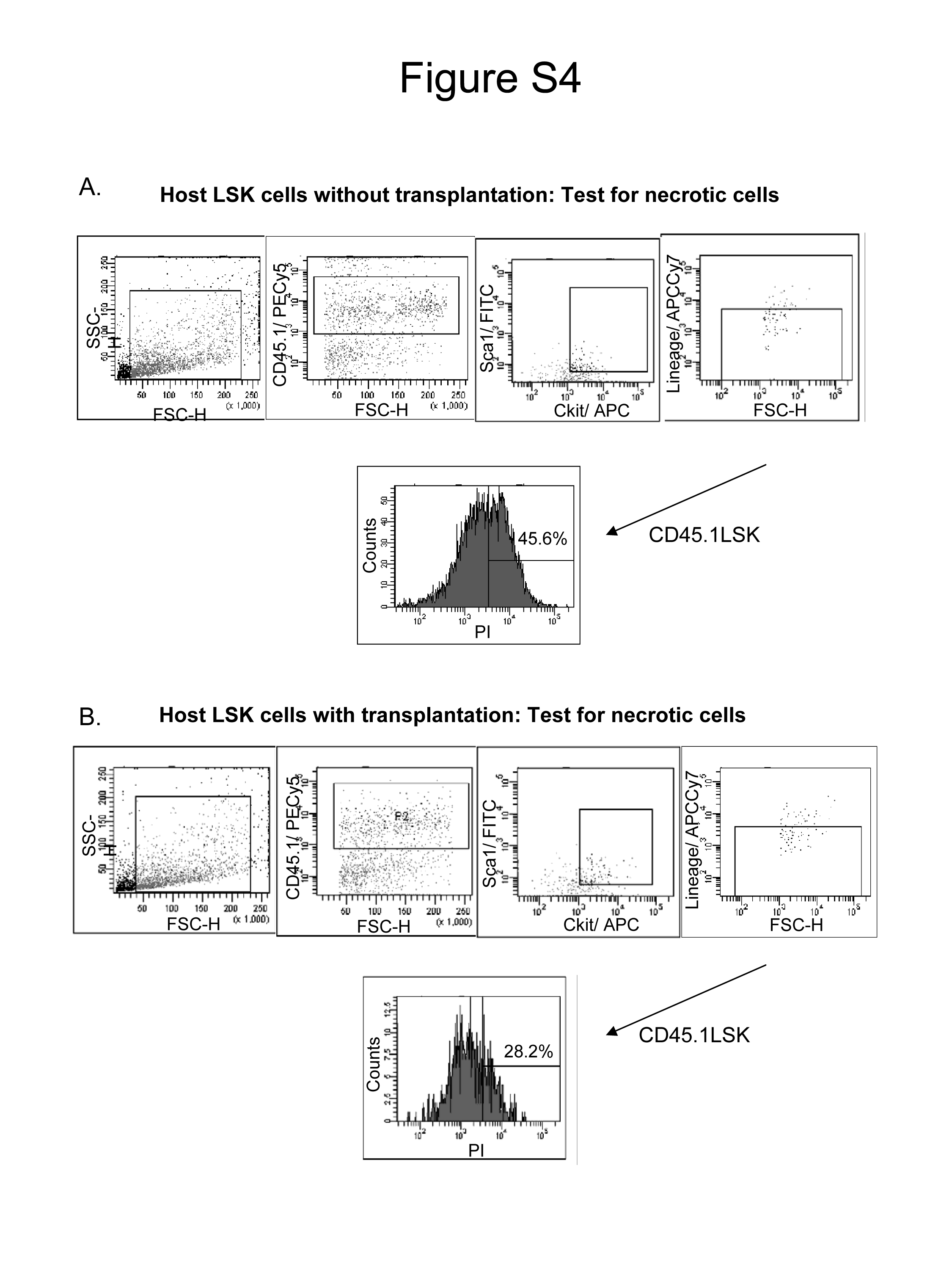

Supplement: Figure S4 — In vivo cytoprotection of irradiated host cells. Sub-lethally irradiated mice were left untransplanted (A) or transplanted (B) with 3×10^4^ CD45.2LSK cells. Mice were sacrificed 3, 5 and 10 days of transplantation and host LSK cells were analyzed for necrotic cells by staining with PI. Representative dot-plots are shown. (TIF) [file pone.0050693.s004.tif]

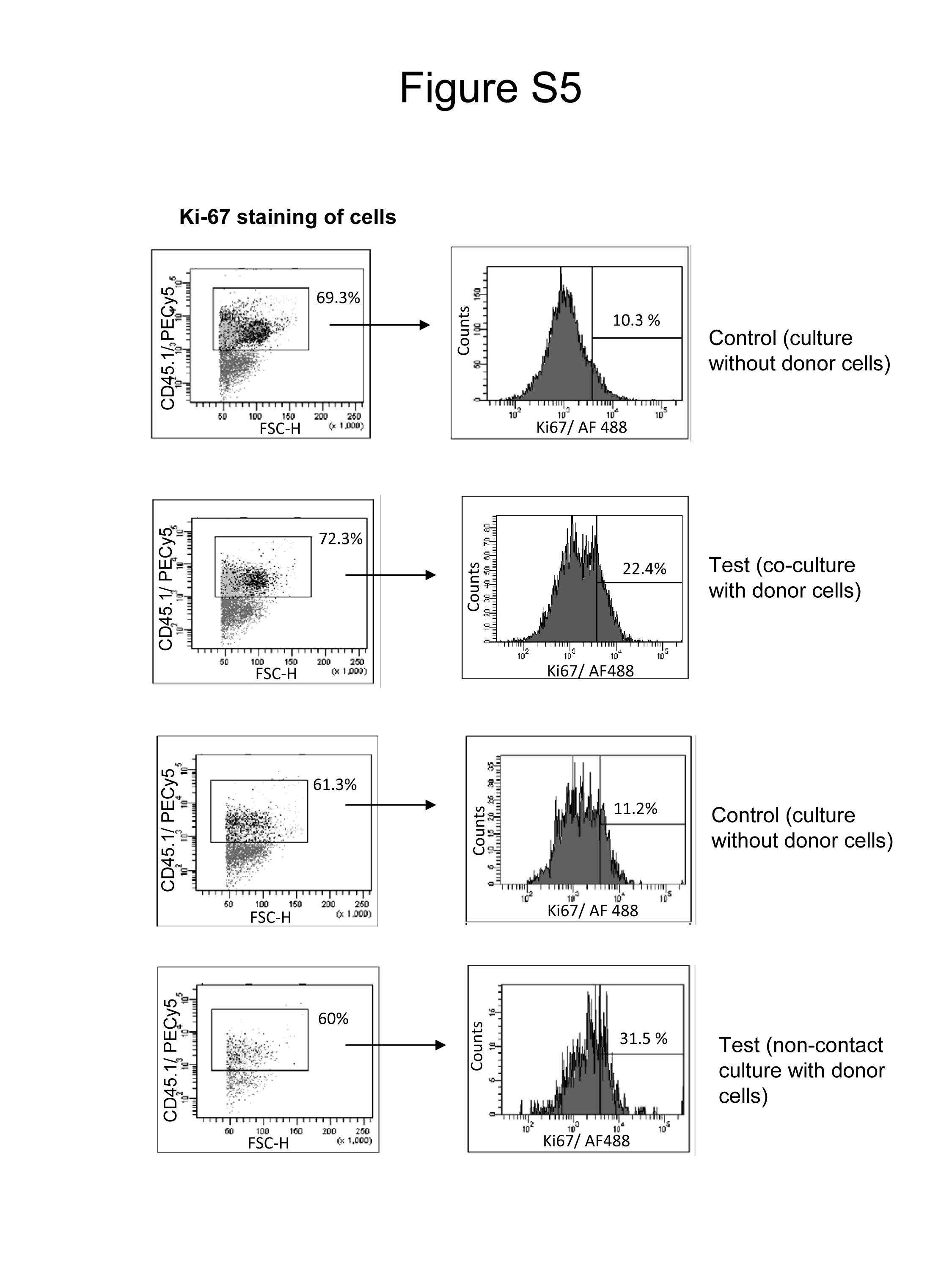

Supplement: Figure S5 — In vitro proliferation of irradiated host cells. Sub-lethally irradiated host (CD45.1) cells were cultured in the absence (control) or in the presence (test) of unirradiated CD45.2+ cells in contact or without contact. The host cells were analyzed for Ki67 staining by flowcytomtery. (TIF) [file pone.0050693.s005.tif]

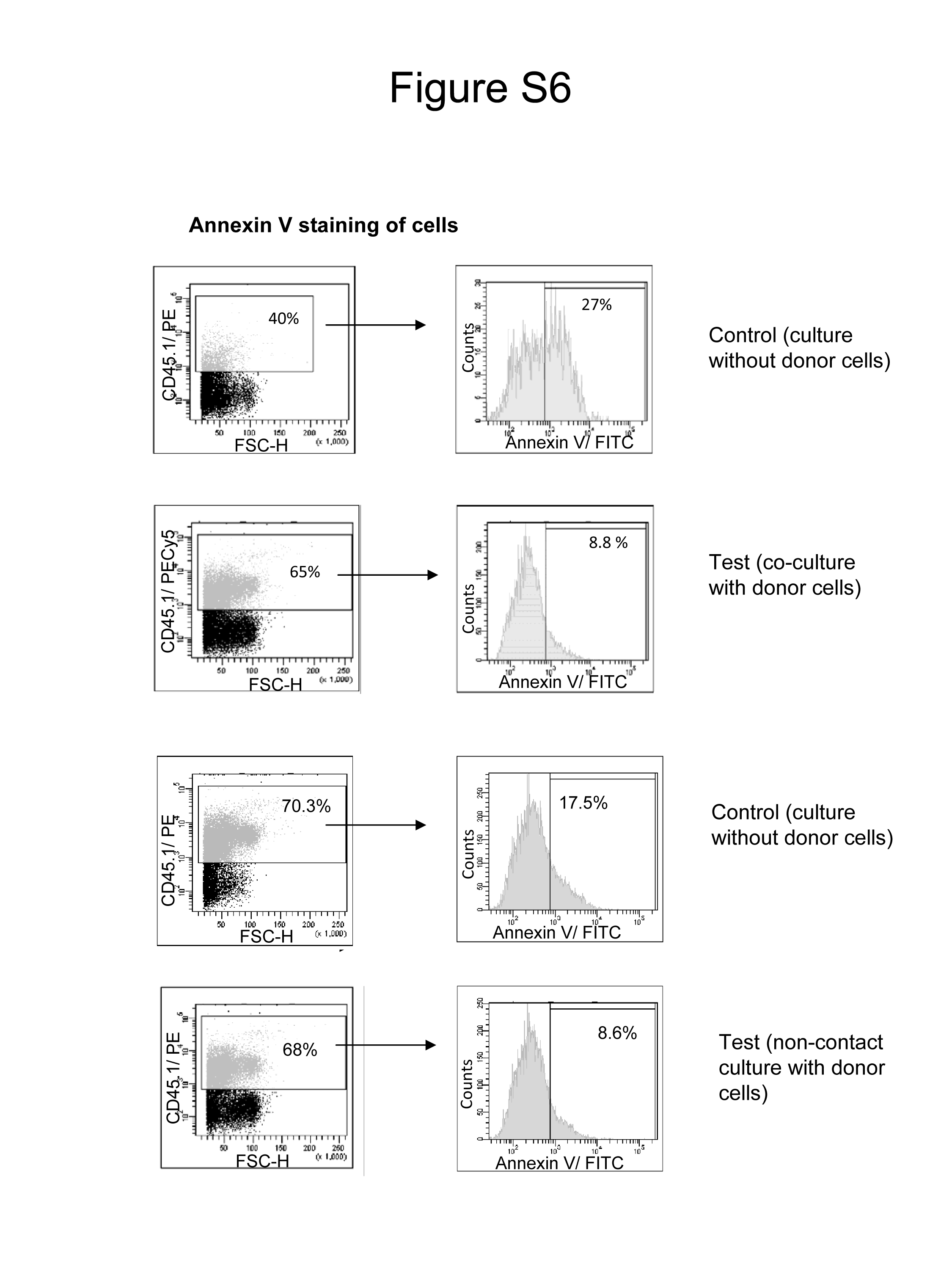

Supplement: Figure S6 — In vitro cytoprotection of irradiated host cells. Sub-lethally irradiated host (CD45.1) cells were cultured in the absence (control) or in the presence (test) of unirradiated CD45.2+ cells in contact or without contact. The host cells were analyzed for Annexin V staining by flowcytomtery. (TIF) [file pone.0050693.s006.tif]

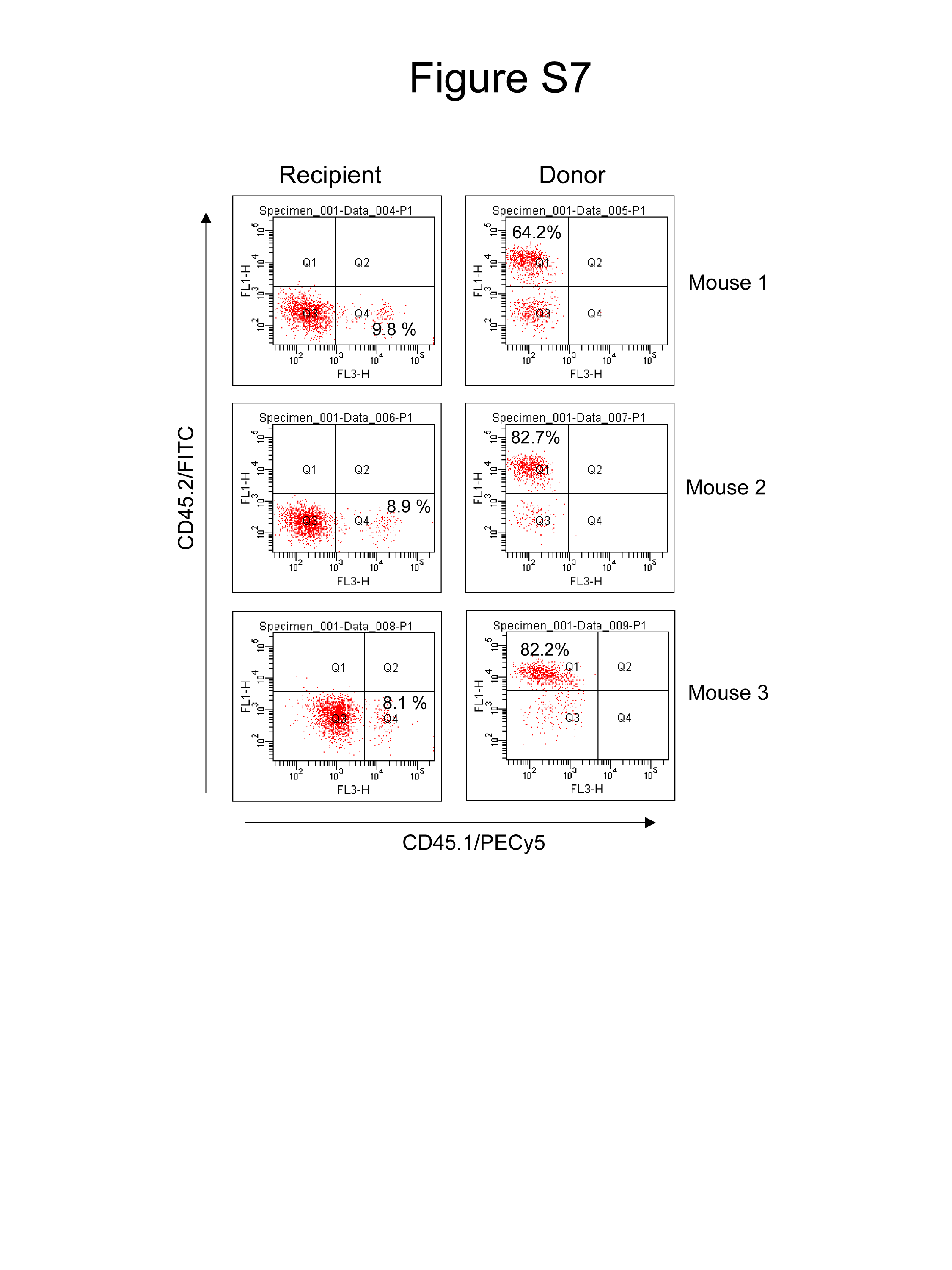

Supplement: Figure S7 — Donor cells chimerism. Mice were transplanted with 10×106 crude donor (CD45.2) cells. Chimerism was determined seven months of transplantation. Dot-plot analyses show chimerism of donor (CD45.2) and host (CD45.1) cells for three mice (n = 3). (TIF) [file pone.0050693.s007.tif]
