# Supplementary material for: Donor Hematopoietic Stem Cells Confer Long-Term Marrow Reconstitution by Self-Renewal Divisions Exceeding to That of Host Cells
Source: PLoS One. 2012 Dec 5;7(12):e50693. doi: 10.1371/journal.pone.0050693 (PMC3515605; doi:10.1371/journal.pone.0050693)
Supplement: Table S1 — Real-time primers sequences, amplicon sizes, annealing temperatures and cycle number of PCR reactions. (DOC) [file pone.0050693.s008.doc]

**Table S1:** Real-time primers sequences, amplicon sizes, annealing temperatures and cycle number of PCR reactions

| Genes | Primers | Annealing Temp (^o^C) | Cycle | Amplicon  Size (bp) |
| --- | --- | --- | --- | --- |
| *IL6* | GAAATGATGGATGCTACC (F)  GGCTTTGTCTTTCTTGTT (R) | 60 | 40 | 133 |
| *IL3* | ACTGATGATGAAGGACCC (F)  CAGATGTAGGCAGGCAA (R) | 60 | 40 | 151 |
| *Wnt3a* | CACCACCGTCAGCAACA (F)  GCACCCACAGATAGCAGC (R) | 60 | 40 | 150 |
| *Flt3L* | CTGTTGCTGCTGCTGAGT (F)  AAGCAGGTGGTCAGTCAA (R) | 60 | 40 | 113 |
| *VEGF* | CCTTCGTCCTCTCCTTACCC (F)  AAGCCACTCACACACACAGC (R) | 60 | 40 | 117 |
| *Jag2* | CCTGGCGGGGCATGTAGAGT (F)  CCAGGCAGGCTAACGCAGTG (R) | 60 | 40 | 118  100 |
| *SCGF* | CACAAGTGCTTCCTGCTCTC (F)  AGTACCGGCTTAGCGCATC (R) | 60 | 40 |
| *SCF* | TGTTCTTGCTACCCGTGACCT (F)  CCTCCAGAGTCCTGCTCCG (R) | 60 | 40 | 127  122 |
| *GAPDH* | ACGGCCGCATCTTCTTGTGCA (F)  CAGGCGCCCAATACGGCCAA (R) | 60 | 40 |
| *p21* | CTGGACTGGGCACTCTTGTC (F)  CTCCTACCATCCCCTTCCTC (R) | 60 | 40 | 119 |

F: Forward primer and R: Reverse primer
