# Supplementary material for: Donor Hematopoietic Stem Cells Confer Long-Term Marrow Reconstitution by Self-Renewal Divisions Exceeding to That of Host Cells
Source: PLoS One. 2012 Dec 5;7(12):e50693. doi: 10.1371/journal.pone.0050693 (PMC3515605; doi:10.1371/journal.pone.0050693)
Supplement: Method S1 — Real-time RT-PCR. (DOCX) [file pone.0050693.s009.docx]

**Methods S1.**

**Real-time RT-PCR:** Total RNA was recovered using TRI Reagent^TM^ (Sigma) according to the manufacturer’s instructions. cDNA was synthesized from 0.5 μg total RNA using the ProtoScript First Strand cDNA Synthesis Kit (NEB, Beverly, MA) and was subjected to PCR amplification with primers selective for target gene segments. Real-time RT-PCR was performed with the Master Cycler ep Realplex 4 (Eppendorf AG, Hamburg, Germany) according to the manufacturer’s instructions. Real-time RT-PCR was carried out in triplicates of each 10 µl reaction volume containing 5 µl 2X *Power* SYBR® Green PCR Master Mix (Applied Biosystems), 0.5 µM of each primer, and 1 µl of cDNA. For normalization, the expression level of the housekeeping gene (GAPDH) was measured as an endogenous control. Fold gene expression in test samples (day 1, 2, 3, 5, 15) were determined by comparing test values with that of the control mice (unirradiated and untransplanted). The 2^-ΔΔCt^ value (relative) was calculated using Realplex 2.2 software (Eppendorf) and expressed as fold change.
